# Machine learning-based segmentation of the rodent hippocampal CA2 area from Nissl-stained sections

**DOI:** 10.3389/fnana.2023.1172512

**Published:** 2023-06-28

**Authors:** Yuki Takeuchi, Kotaro Yamashiro, Asako Noguchi, Jiayan Liu, Shinichi Mitsui, Yuji Ikegaya, Nobuyoshi Matsumoto

**Affiliations:** ^1^Graduate School of Pharmaceutical Sciences, The University of Tokyo, Tokyo, Japan; ^2^Department of Rehabilitation Sciences, Graduate School of Health Sciences, Gunma University, Maebashi, Gunma, Japan; ^3^Institute for AI and Beyond, The University of Tokyo, Tokyo, Japan; ^4^Center for Information and Neural Networks, National Institute of Information and Communications Technology, Osaka, Japan

**Keywords:** hippocampus, CA2, RGS14, Nissl, U-Net, machine learning, mouse, prairie vole

## Abstract

The hippocampus is a center of learning, memory, and spatial navigation. This region is divided into the CA1, CA2, and CA3 areas, which are anatomically different from each other. Among these divisions, the CA2 area is unique in terms of functional relevance to sociality. The CA2 area is often manually detected based on the size, shape, and density of neurons in the hippocampal pyramidal cell layer, but this manual segmentation relying on cytoarchitecture is impractical to apply to a large number of samples and dependent on experimenters’ proficiency. Moreover, the CA2 area has been defined based on expression pattern of molecular marker proteins, but it generally takes days to complete immunostaining for such proteins. Thus, we asked whether the CA2 area can be systematically segmented based on cytoarchitecture alone. Since the expression pattern of regulator of G-protein signaling 14 (RGS14) signifies the CA2 area, we visualized the CA2 area in the mouse hippocampus by RGS14-immunostaining and Nissl-counterstaining and manually delineated the CA2 area. We then established “CAseg,” a machine learning-based automated algorithm to segment the CA2 area with the F1-score of approximately 0.8 solely from Nissl-counterstained images that visualized cytoarchitecture. CAseg was extended to the segmentation of the prairie vole CA2 area, which raises the possibility that the use of this algorithm can be expanded to other species. Thus, CAseg will be beneficial for investigating unique properties of the hippocampal CA2 area.

## 1. Introduction

The hippocampus plays a pivotal role in memory (Scoville and Milner, 1957; Victor and Agamanolis, 1990; Squire and Zola-Morgan, 1991; Kol et al., 2020), learning (Schapiro et al., 2017; Kragel et al., 2021), and spatial navigation (O’Keefe and Dostrovsky, 1971; Brun et al., 2002) and is anatomically divided into three subfields: the CA1, CA2, and CA3 areas (van Strien et al., 2009; Matsumoto et al., 2019). Among these three subdivisions, the CA2 area is distinct from the CA1 and CA3 areas in terms of functional relevance to sociality (Hitti and Siegelbaum, 2014; Stevenson and Caldwell, 2014; Oliva et al., 2020; Rey et al., 2022) and aggression (Pagani et al., 2015; Leroy et al., 2018). Previous studies have also suggested that the CA2 area is associated with psychiatric disorders such as autism spectrum disorder (Modi et al., 2019), manic depression (Benes et al., 1998, 2001), schizophrenia (Benes et al., 1998, 2001), and temporal lobe epilepsy (Whitebirch et al., 2022).

To better understand the unique properties of the CA2 area in physiology and pathology (Dudek et al., 2016), reliable methods for the segmentation of the CA2 area are required. Researchers often manually map the areas of the hippocampal formation in humans (Amunts et al., 2005; Amunts and Zilles, 2015) and rodents (Tzakis and Holahan, 2019) based on neuronal size, shape, and density. For example, in rodents, CA2 pyramidal neurons are similar to CA3 neurons in that their somata are larger and less densely packed than those of CA1 neurons (Lorente de Nó, 1934; Ishizuka et al., 1995), but CA2 pyramidal neurons are differentiated from CA3 pyramidal neurons in terms of apical dendritic branching (Ishizuka et al., 1995; Helton et al., 2019). Moreover, CA2 neurons lack thorny excrescences on their proximal dendrites, unlike typical CA3 neurons (Ishizuka et al., 1995); note that a small population of CA3 pyramidal neurons is athorny (Hunt et al., 2018), whereas some CA2 pyramidal neurons are thorny (Radzicki et al., 2023). As for the rodent CA2 area, such manual segmentation that relies on cytoarchitecture is valid to some extent (Tzakis and Holahan, 2019), but still, impractical when applied to a large number of samples due to time constraints. In addition, the criteria for the manual segmentation are dependent on experimenters’ proficiency and are prone to their bias (Powell et al., 2008). Recently, the CA2 area has been redefined based on gene expression (Lein et al., 2005), leading to the discovery and usage of molecular markers for the CA2 area, such as RGS14, STEP, and PCP4 (Kohara et al., 2014; Matsumoto et al., 2016; Noguchi et al., 2017; Pang et al., 2019). Less biased and more accurate identification of the rodent CA2 area requires immunohistochemistry (Hitti and Siegelbaum, 2014; Kay et al., 2016; Leroy et al., 2018; Meira et al., 2018; Brown et al., 2020; Whitebirch et al., 2022), but it takes more days to complete immunohistochemistry than Nissl-staining only. Thus, we questioned whether the CA2 area could be segmented more reliably and automatically based on cytoarchitecture alone.

To this end, we first immunostained RGS14-positive neurons in the mouse hippocampus together with Nissl-counterstaining, and manually delineated the CA2 area with the aid of RGS14-immunofluorescence. We then attempted to implement a machine learning-based algorithm to segment the CA2 area in Nissl-counterstained images that visualized cytoarchitecture alone; we coined “CAseg” for this algorithm. We further investigated whether CAseg could be extended to brain sections of prairie voles, rodents as small as mice.

## 2. Materials and methods

### 2.1. Data acquisition

#### 2.1.1. Animal ethics

Animal experiments were performed with the approval of the animal experiment ethics committee at the University of Tokyo (approval numbers: P29–15 and P3-1) and in accordance with the University of Tokyo guidelines for the care and use of laboratory animals. The experimental protocols were in accordance with the Fundamental Guidelines for the Proper Conduct of Animal Experiments and Related Activities in Academic Research Institutions (Ministry of Education, Culture, Sports, Science and Technology, Notice No. 71 of 2006), the Standards for Breeding and Housing of and Pain Alleviation for Experimental Animals (Ministry of the Environment, Notice No. 88 of 2006) and the Guidelines on the Method of Animal Disposal (Prime Minister’s Office, Notice No. 40 of 1995).

A total of fourteen animals were housed in groups (unless otherwise specified) under conditions of controlled temperature and humidity (22 ± 1°C, 55 ± 5%) and maintained on a 12 h:12 h light/dark cycle with ad libitum access to food and water. All efforts were made to minimize animal suffering.

#### 2.1.2. Histology

Twelve young adult (4–6 weeks old) male ICR mice (Japan SLC, Japan) were anesthetized via intraperitoneal administration of 150 mg/ml urethane dissolved in saline; two adult prairie voles, a gift from Dr. Shinichi Mitsui at Gunma University, were anesthetized with 2–3% isoflurane followed by urethane. Anesthesia was confirmed by the lack of reflex responses to tail and toe pinches. The mice and prairie voles were transcardially perfused with chilled 0.01 M phosphate-buffered saline (PBS) followed by 4% paraformaldehyde in PBS. The animals were then decapitated, and their brains were carefully removed. These brains were postfixed in 4% paraformaldehyde overnight and washed with PBS three times for 10 min each. Serial coronal sections were prepared using a vibratome at a thickness of 100 μm from the anterior region to the posterior region; that is, the 100-μm-thick coronal sections were continuously picked up and collected.

Basic immunohistochemistry procedures have been described previously (Matsumoto et al., 2016; Noguchi et al., 2017; Kashima et al., 2019; Liu et al., 2021). Sections were blocked with 10% goat serum and 0.3% Triton X-100 in PBS for 1 h at room temperature and incubated with a mouse primary antibody against RGS14 (1:500, 75–170, NeuroMab, CA, USA) for 16 h at 4°C. Sections were washed with PBS three times for 10 min each and then incubated with Alexa Fluor 488-conjugated goat secondary antibody against mouse IgG (1:500, A11029, Invitrogen, MA, USA) and NeuroTrace 435/455 blue fluorescent Nissl stain (1:500, N21479, Thermo Fisher Scientific, MA, USA; hereafter, Nissl) for 6 h at room temperature, followed by another three 10-min washes with PBS.

#### 2.1.3. Confocal imaging

Before images of each set of slices were captured, the laser power for each fluorescence channel was set just below the intensity that would cause fluorescence saturation (Yamashiro et al., 2021). The images (1024 × 1024 pixels, 16-bit intensity) were acquired at a Z-interval of 1.0 μm using an FV1200 confocal microscope (Olympus, Tokyo, Japan) equipped with 20× objectives and Z-stacked using ImageJ software (National Institutes of Health, MD, USA). Note that all images used in this study did not contain any portion of the dentate gyrus.

### 2.2. Data analysis

The images were processed and analyzed using ImageJ software and Python 3. The summarized data are presented as the mean ± the standard deviation unless otherwise specified. *P* < 0.05 was considered statistically significant.

#### 2.2.1. Preprocessing

Images that contained clearly defined cell shapes and minimal staining irregularities were selected by skilled experimenters since poor-quality input images (i.e., without any features to be learned) usually degrade the performance of subsequent machine learning and deep learning. Images containing the entire CA2 area and parts of the CA1 and CA3 areas were used as training data so that the current deep learning scheme could learn the difference between the CA2 area and other (i.e., CA1 and CA3) areas.

**FIGURE 1:**
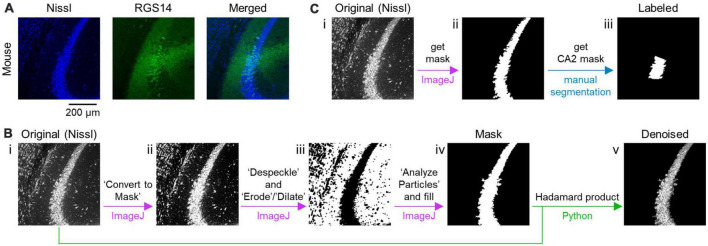
Preprocessing of fluorescent images of the CA2 and surrounding areas in the hippocampus. **(A)** Representative images of a section of the mouse hippocampus immunostained with RGS14 (green, middle) and counterstained with Nissl (blue, left). A merged image is also shown (right). **(B)** Workflow of denoising the original Nissl-stained image using custom-written scripts. An original image is binarized using a command in ImageJ, which is specified by single quotes (i–ii). The binarized image is despeckled and eroded/dilated (ii–iii). Then, a mask image is created (iii–iv). Using the mask image, the original image is denoised (iv–v). Each arrow in purple indicates transformation from a previous image to the next one using ImageJ, while green arrows signify generation of a denoised image from masked and original images based on a principle of Hadamard product using Python. **(C)** Workflow of creating a labeled image of the CA2 area. A mask image of the pyramidal cell layer is created from the original image (i–ii) in the similar manner as panel **(B)** and then manually segmented by skilled experimenters to create a mask image that labels the CA2 area (ii–iii).

Using ImageJ, the images in the stacks were extracted, and the brightness and contrast were adjusted by a tool accessed through *Image > Adjust > Brightness/Contrast > ‘Auto’*. The color channels were split, and two separate channels (e.g., RGS14 and Nissl) of images were thereby obtained (Figure 1A). Consequently, a total of 295 images from 16 slices from 9 mice were used (Supplementary Table 1).

To remove noise that might interfere with machine learning, the area of the pyramidal cell layer was roughly defined as follows. Using an ImageJ tool accessible at *Process > Binary > Convert to Mask*, the Nissl images were roughly binarized; note that in some cases, the Nissl images were further processed with a threshold of Nissl fluorescence preset automatically by a tool available at *Image > Adjust > Threshold > Auto* (Figure 1B, i–ii). The binarized images were despeckled (by a tool accessible at *Process > Noise > Despeckle*) and eroded/dilated (using a tool accessed through *Process > Binary > Erode* or *Dilate*) for denoising (Figure 1B, ii–iii). The largest contour was gleaned and applied to the original image to remove all particles (i.e., noise) outside the contour to create a mask image (Figure 1B, iii–iv). Using a custom-written Python script, all objects outside the segmented image (obtained by the process above) were removed from the corresponding Nissl image in such way that each pixel value (i.e., 0, 1, …, 255) in the original Nissl image was either transformed into 0 or preserved when each corresponding pixel value in the mask image was 255, respectively. The noise removal was based on the principle of the Hadamard product of a matrix and another binary matrix (containing 0 or 1) that represents the mask image (Figure 1B, iv–v). These preprocessing procedures have been streamlined by custom-made Python (i.e., Jython and CPython) routines available at https://x.gd/BPFxj.

Image segmentation masks of the CA2 area (i.e., masked images) were next created manually and defined as labeled data. Our manual algorithm was first run to label the pyramidal cell layer of the hippocampus in a manner similar to the procedure illustrated in Figure 1B. Three skilled professionals independently identified and manually annotated the CA2 area from the corresponding fluorescence image of Nissl-staining and RGS14-immunostaining. Only images including the CA2 area agreed upon by all professionals were used as labeled data (Figure 1C). Note that the three skilled experimenters determined the CA2 area based not only on the fluorescence image of RGS14 immunofluorescence images but also on the images of the Nissl-stained cell bodies.

As for Figure 5, two separate channels for RGS14 and Nissl staining were utilized to manually identify both the pyramidal cell layer and the CA2 area, after which all noise outside the pyramidal cell layer was eliminated in the similar way as illustrated in Figure 1 (see also Supplementary Table 1). Image segmentation masks of the CA2 area were then created. Note that the aim was to see if the same model trained using slices of mouse brains can be used for slices of prairie vole brains.

#### 2.2.2. U-Net architecture

**FIGURE 2:**
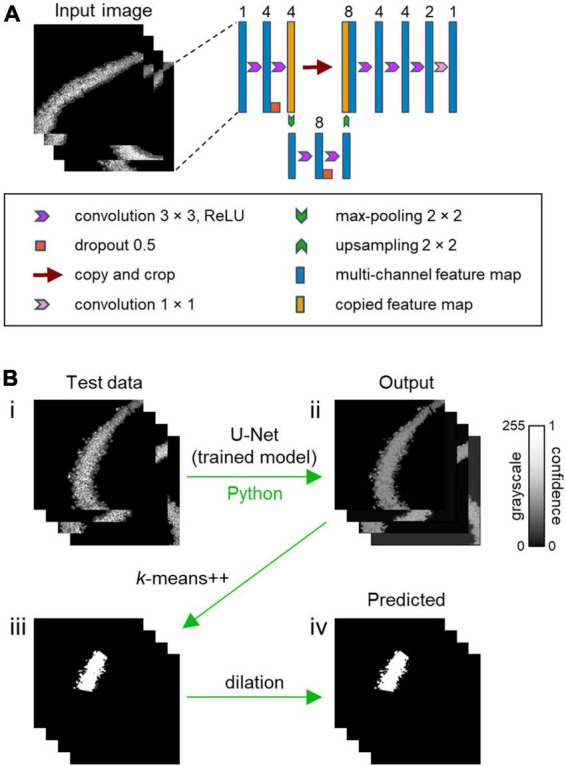
CAseg: a machine learning-based automated algorithm for segmentation of the hippocampal CA2 area. **(A)** U-Net architecture. The U-Net algorithm processes the input images of the hippocampus (left), which are the outputs of preprocessing (see Figure 1B, v). The algorithm then trains the model using corresponding labeled images (see Figure 1C, iii). The number of channels is denoted above the blue and orange boxes. **(B)** Test image data are applied to the trained model to obtain output images (i–ii), which are roughly transformed into segmentation mask images using *k*-means++ (ii–iii). The rough segmentation masks are finally dilated to identify the CA2 area (i.e., predicted images).

The U-Net-based model in our algorithm contained the following layers: (i) convolution and (ii) dropout (Figure 2A; Long et al., 2014; Ronneberger et al., 2015; Yamashiro et al., 2021).

(i) In the process called convolution, kernels were slid over the input vector, and the result of multiplying the kernel’s own values with those of the input data is output to the next layer, called feature maps (O’Shea and Nash, 2015). The convolutional layer surrounded a feature matrix with zero (i.e., zero padding) so that the size of the inputs and outputs would be equal. Then, a rectified linear unit (i.e., ReLU), expressed as the following function (Agarap, 2018), f(x) = max(0, x), was adopted. Since there were multiple patterns of output filters in the convolution, multichannel feature maps were obtained in this process.
(ii) Deep learning algorithms often come with a problem called overfitting, in which the model adapts extremely well to the training data but does not perform well on test data. Overfitting often occurs when the model is trained using less-common data or puts excessive focus on complicated relationships in the input data. To avoid overfitting, dropout, a method in which artificial neurons are randomly omitted from the neural network, was utilized. The probability that each unit would be retained was 0.5 in our model (Srivastava et al., 2014; dos Santos and Papa, 2022).

The weight of the present model was initialized (He et al., 2015), giving adequate consideration to the characteristics of ReLU. The contracting path (Figure 2A, *left*) applies two 3 × 3 convolutions, followed by dropout and 2 × 2 max-pooling with stride 2 for downsampling. After downsampling, the number of feature channels was doubled, and the same process was performed for the subsequent input matrix. The expansive step (Figure 2A, *right*) required a 2 × 2 convolution for upsampling to halve the number of feature channels. The output after upsampling was coupled on the correspondingly cropped feature map from the contracting path, succeeded by three 3 × 3 convolutions. The final 1 × 1 convolution layer employed a sigmoid activation function, and the number of channels finally turns to 1, matching that of the true label (Ronneberger et al., 2015). The U-Net model was implemented using Keras, a Python deep learning library, and the TensorFlow backend.

#### 2.2.3. Deep learning-based algorithm in CAseg

After preprocessing, the U-Net model was implemented to characterize the CA2 area (Figure 2A), and the most plausible areas were extracted. The neural network was optimized by adaptive moment estimation (i.e., Adam) with a learning rate of 0.001. The other parameters were as follows: *beta_1* (i.e., an exponential decay rate for the first moment estimates) = 0.9, *beta_2* (i.e., an exponential decay rate for the second moment estimates) = 0.999, and *epsilon* = 1e-7.

In the deep learning scheme of CAseg, to prevent similar images from being utilized in learning, 295 pairs of Nissl-stained fluorescence images and image segmentation masks of the CA2 area were shuffled without changing the combination of the pairs, after which the data were split into five subgroups (*S*_1_, *S*_2_, *S*_3_, *S*_4_, and *S*_5_). The pairs of images in one of the five subgroups (e.g., *S*_1_) were chosen as test data, while all other images included in the rest of the subgroups the rest of the subgroups (e.g., *S*_2_, *S*_3_, *S*_4_, and *S*_5_) were fed into the U-Net model as training data. This procedure was repeated for all *S*_*i*_ (*i* = 1, 2, 3, 4, 5) so that five models would be created in total. In the training session, 20% of the training data were used as validation data, and a pair was extracted from the rest of the data. The model was trained with the pair, and binary cross-entropy loss was calculated from predicted images and the corresponding true labels. The model was trained to minimize the loss value. The same process was repeated for 100 epochs. The model was saved or overwritten exclusively when the loss was minimized. Note that the training data were shuffled before each epoch. The model training was performed on a GeforceRTX2080Ti GPU (Nvidia, CA, USA), and it took approximately 1 h to complete the training.

#### 2.2.4. *k*-means++ algorithm in CAseg: postclustering of the hippocampal subfields

After the prediction of a test dataset by the trained U-Net (Figure 2B, i), the intensity of each pixel in the “output” images ranged between 0 and 1 (Figure 2B, ii). Higher values indicated stronger confidence that the pixels were inside the CA2 area (Figure 2B, ii). Thus, a conventional machine learning method called “*k*-means++” was additionally implemented (Arthur and Vassilvitskii, 2007); this method separates the given data in an unsupervised manner into the desired number of clusters based on the relationship among pixel coordinates, using scikit-learn, a Python machine learning library. This method enabled us to identify the CA2 area. The parameters for *k*-means++ were as follows: *n_int* (i.e., the number of times when the *k*-means++ algorithm was run with different centroid seeds) = 10, *max_ite* (i.e., the maximum number of iterations of the *k*-means++ algorithm for a single run) = 300, *tol* (i.e., relative tolerance with regards to the Frobenius norm of the difference in the cluster centers of two consecutive iterations to declare convergence) = 1e-4, and *algorithm* (i.e., *k*-means++ algorithm to use) = “llyoid.”

For a given output image, an “intensity threshold” was set at 20, 30, 40, or 50% of the highest intensity value among all pixels, after which pixel values lower than the threshold were translated into nil. The translated images were eroded twice with a 3 × 3 kernel to exclude tiny clusters. The eroded images were processed with *k*-means++ (Figure 2B, ii–iii); note that there should be three clusters in *k*-means++, because the hippocampal pyramidal cell layer was divided into the CA1, CA2, and CA3 areas. Then, the predicted image segmentation of the CA2 area was produced by dilation with a 2 × 2 kernel (Figure 2B, iii–iv). This dilation was iterated once, twice, or three times, or no dilation was performed (i.e., zero iteration) (Figure 4 and Supplementary Tables 2–5).

Since there were thresholds of 20, 30, 40, and 50%, and the number of iterations in the dilation process was four (see above), there were 16 patterns to validate (Figure 4 and Supplementary Tables 2–5). Among these patterns, the model that yielded the highest F1-score (i.e., in the case of a threshold of 30% fluorescence intensity and 1 iteration) was utilized to predict the CA2 area of new slices from mice and prairie voles (Figure 5).

To assess the performance of our machine learning-based algorithm, surrogate data (i.e., incorrect CA2 labels for the fluorescence images of Nissl staining) were created by shuffling the 295 pairs of fluorescence images of Nissl staining and corresponding image segmentation masks of the CA2 area. These images were applied to our machine learning-based algorithm to evaluate performance scores of randomly labeled data.

#### 2.2.5. Evaluation of model performance

The performance of the model was evaluated using the precision, recall, IoU (intersection over union) and F1-score (Rezatofighi et al., 2019; Orita et al., 2020; Ogasawara et al., 2021; Yamashiro et al., 2021; Ottom et al., 2022). These metrics were defined as follows:


$$p\,r\,e\,c\,i\,s\,i\,o\,n=\frac{T\,P}{T\,P+F\,P}$$

$$r\,e\,c\,a\,l\,l=\frac{T\,P}{T\,P+F\,N}$$

$$I\,o\,U=\frac{T\,P}{T\,P+F\,P+F\,N}$$

where TP, FP, and FN represent the true positive, false positive, and false negative, respectively. The F1-score was calculated as the harmonic mean of the precision and recall.

## 3. Results

We immunostained coronal sections of the mouse brain against RGS14, a CA2 marker protein (Evans et al., 2014; Kohara et al., 2014) and counterstained them with Nissl stain (Figure 1A), detecting abundant RGS14-immunoreactive cells (i.e., putative CA2 neurons) in the hippocampal pyramidal cell layer for all 12 mice tested (Figure 1A).

We denoised the images by removing all objects outside the pyramidal cell layer for our machine learning scheme (Figure 1B) to obtain segmentation masks of the pyramidal cell layer. Based on fluorescence images of Nissl and RGS14 staining, skilled experimenters manually delineated the CA2 area from the segmentation masks of the pyramidal cell layer; this delineated area was defined thereafter as ground truth (Figure 1C).

**FIGURE 3:**
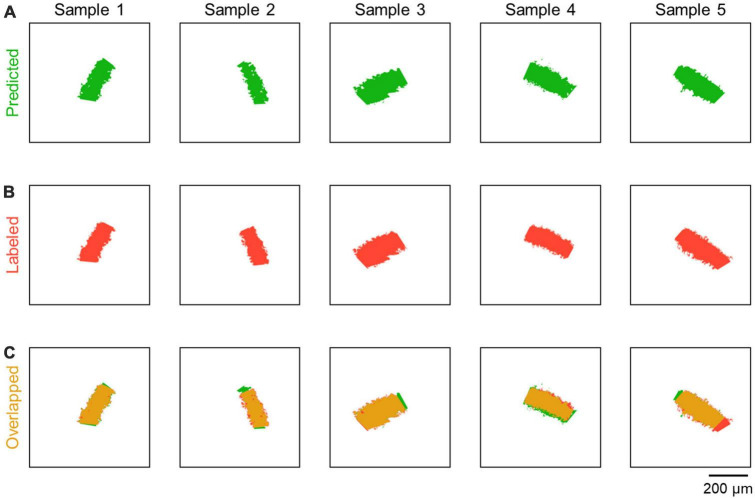
Comparison between predicted and labeled images. **(A)** Representative images predicted by CAseg (green). **(B)** Images labeled as the CA2 area (red; see Figure 1C, iii), each of which corresponds to the predicted image (green) in the same column. **(C)** Images showing the overlap between the predicted and labeled images (yellow). Note that the images of the hippocampi of both hemispheres were randomly selected.

For CAseg, we first utilized U-Net, a type of deep neural network that has been trained and has learned to make dense predictions for per-pixel tasks using fewer images for more accurate segmentations (Long et al., 2014; Ronneberger et al., 2015; Yamashiro et al., 2021). We fed these images [i.e., the denoised pyramidal cell layer (Figure 1B, v) and the segmentation mask of the CA2 area (Figure 1C, iii)] into U-Net for training (Figure 2A). The trained models labeled pixels in the test image with continuous values between 0 and 1 (i.e., the probability of being included in the CA2 area); however, our U-Net-based algorithm labeled the pyramidal cell layer not only in the CA2 area but also in the CA1 and CA3 areas (Figure 2B). Thus, we further implemented *k*-means++ and subsequent dilation to automatically isolate the CA2 pyramidal cell layer from the U-Net-predicted images (Figures 2B, 3).

**FIGURE 4:**
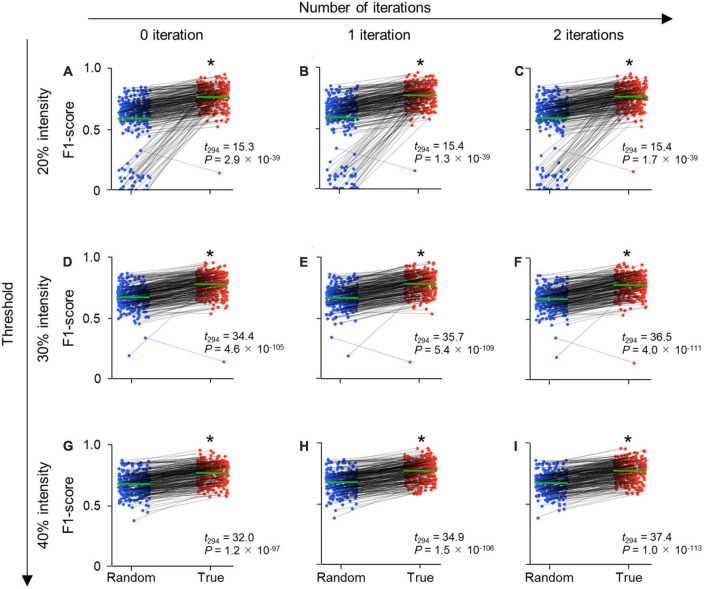
Parameter-independent high performance metrics of CAseg. Each row displays the threshold (%) of fluorescence intensity, while each column indicates the number of iterations of dilation. **(A)** F1-score of CAseg with a 20% threshold and no dilating iteration. The test images were predicted by CAseg trained on random labels (blue) and true labels (red). Each pair of points connected by a line (black) signifies the same test image. The average F1-score of all test data is shown in green. **(B)** The same as panel **(A)**, but for 20% and one iteration. **(C)** The same as panel **(A)**, but for 20% and two iterations. **(D)** The same as panel **(A)**, but for 30% and no iteration. **(E)** The same as panel **(A)**, but for 30% and one iteration. **(F)** The same as panel **(A)**, but for 30% and two iterations. **(G)** The same as panel **(A)**, but for 40% and no iteration. **(H)** The same as panel **(A)**, but for 40% and one iteration. **(I)** The same as panel **(A)**, but for 40% and two iterations. Statistics are compiled by paired *t*-tests (*n* = 295 images; Supplementary Table 4). *P < 0.05.

**FIGURE 5:**
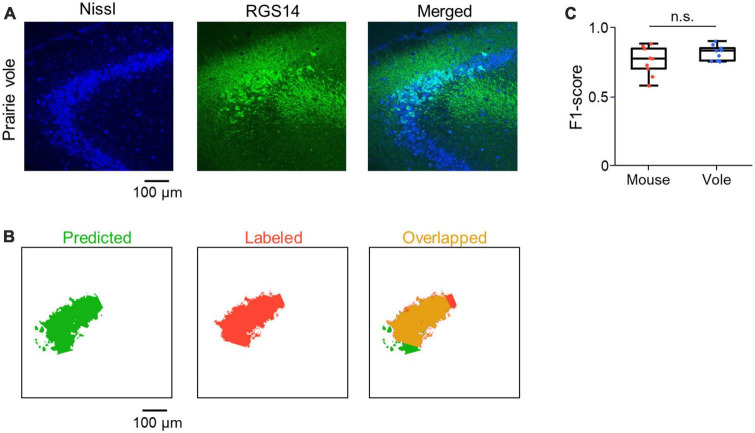
Application of CAseg to the prairie vole hippocampus. **(A)** Representative image of a section of the prairie vole hippocampus immunostained for RGS14 (green) and counterstained with Nissl stain (blue). **(B)** Representative images of the prairie vole CA2 area predicted by CAseg (green, left). Images labeled as the CA2 area (red, middle), each of which corresponds to the predicted image (green). Images showing the overlap between the predicted and labeled images (yellow, right). **(C)** The F1-scores of CAseg predicting test images of mice (red) and prairie voles (blue). Each point represents the F1-score of one image.

To evaluate the segmentation performance of CAseg, we calculated the performance metrics of the F1-score, recall, precision, and IoU on the pixelwise segmentation data. Since these metrics might have been vulnerable to the parameters of the fluorescence intensity threshold and the number of iterations of dilation (see Section “2. Materials and methods”), we calculated the four types of metrics for different combinations of the parameters (i.e., intensity threshold: 20, 30, 40, or 50%; the number of dilation iterations: 0, 1, 2, or 3), confirming that the performance metrics for models on true labels were significantly better than those for models on random labels for each of the 16 cases (Figure 4 and Supplementary Tables 2–5). In particular, we found that an intensity of 30% and one iteration maximized the F1-score (Figure 4 and Supplementary Table 4). This best model had significantly higher F1-scores when trained on true labels than random labels for the images of the rostral, intermediate, and caudal hippocampus. This suggests that CAseg segments the CA2 area, irrespective of the rostrocaudal level of sections (Supplementary Figure 1), although it is still unclear what characteristics (e.g., the curvature and thickness of the pyramidal layer, and the shapes and sizes of cells) in the Nissl images were being recognized by CAseg. When we divided the denoised hippocampus (Figure 1B, v) into three clusters using only *k*-means++, the F1-score (of *k*-means++ only) was significantly lower than that of CAseg (Supplementary Figure 2), indicating that for CAseg, the U-Net-based algorithm is necessary for the accurate segmentation of the CA2 area.

To investigate whether CAseg optimized for the brain sections of mice was also applicable to those of small rodents similar to mice, we prepared 100-μm-thick coronal sections from adult prairie voles in a similar manner to mice and applied CAseg to the vole brain sections. We found RGS14-immunoreactive neurons in the hippocampus of prairie voles (Figure 5A) and acquired 9 images from 9 new stacks each in mice and prairie voles. For a given image from each animal, we predicted the CA2 area with five pretrained deep learning models of CAseg and obtained five F1-scores, which we then averaged. We repeated this calculation for all 18 images (i.e., 9 images from mice and 9 from prairie voles) and found almost the same F1-score in voles as in mice [0.76 ± 0.10 (mouse) vs. 0.82 ± 0.05 (prairie vole)], suggesting that CAseg was also applicable to the vole hippocampus (*P* = 0.13, *t*_16_ = 1.61, *n* = 9 and 9 images from 5 mice and 2 prairie voles, respectively, Student’s *t*-test; Figures 5B, C).

## 4. Discussion

CAseg, the machine learning-based algorithm established in this study, identified the CA2 area in Nissl-stained sections from mice. Using CAseg, we segmented the hippocampal pyramidal cell layer and delineated the CA2 area at a high level of performance. Moreover, our model that was trained on mice enabled us to identify the CA2 area of prairie voles as well.

CAseg requires a relatively small number (∼10^2^) of images as input data to segment the hippocampal CA2 area, compared with ∼10^4^ samples for a general neural network model (Goodfellow et al., 2016). Deep learning alone would not have segmented the CA2 area because of the small datasets in this study. However, we combined deep learning with conventional machine learning in this study to overcome this problem. Indeed, we prepared a limited number of images, in keeping with the 3Rs principle in animal experimentation (replacement, reduction, and refinement) (Maestri, 2021), but this problem was overcome by the combination of U-Net and *k*-means++. We found the high F1-scores for random labels, which we assume resulted from the postclustering by *k*-means++ (Figure 4). When the U-Net-based part of CAseg was trained on the randomly labeled dataset, the U-Net produced incorrect output with relatively low confidence, but *k*-means++ compensated the low confidence to correctly cluster the hippocampal subfields and segment the CA2 area to some extent.

Intuitively, the performance of our models trained on true labels should have varied depending on parameters (i.e., the intensity threshold and the number of iterations of dilation; see Section “3. Results”) during postprocessing because the number of pixel coordinates obtained from a test image was affected by the parameters. Nevertheless, each performance metric (i.e., the precision, recall, F1-score, and IoU) was stable even when tested with various thresholds and iterations (Figure 4), suggesting that CAseg is robust against these parameters and saves time that would have been spent adjusting them, even though the algorithm was created using a small dataset.

The definition of the CA2 area has been controversial. The CA2 area was originally identified and described based on its intrahippocampal and extrahippocampal connectivity such as afferent innervation from the supramammillary nucleus and lack of mossy fiber innervation from the dentate gyrus (Lorente de Nó, 1934; Maglóczky et al., 1994; Kohara et al., 2014; Robert et al., 2018). The rodent CA2 area is also distinguished from the CA1 and CA3 areas in terms of morphology (see Introduction). Recently, the CA2 area has been molecularly defined as a broader region (between the CA1 and CA3 areas) than classically identified (Lein et al., 2005; Kohara et al., 2014). This definition of the CA2 area is visually obvious on histochemical staining for molecular markers such as RGS14 (Kohara et al., 2014; Gerber et al., 2019), PCP4 (Kohara et al., 2014; Alexander et al., 2018), STEP (Kohara et al., 2014; Matsumoto et al., 2016), and Necab-2 (Zimmermann et al., 2013; Gerber et al., 2019). Nevertheless, even if such molecular markers are used, the border between the CA2 and CA1/CA3 areas is difficult to distinguish, sometimes requiring Gaussian blurring to avoid arbitrary segmentation (San Antonio et al., 2014). In this light, when only somata or nuclei are stained (e.g., by using Nissl stains or DAPI), the molecular marker-based definition of the CA2 area is ambiguous at least for humans; this ambiguity may cause experimenter-to-experimenter variability in the definition of the CA2 area. In this study, skilled experimenters manually defined the CA2 area as an RGS14-positive region. In Nissl-stained sections, this molecularly isolated region was indistinguishable from the CA1 and CA3 areas for humans, but our computerized algorithm, CAseg, precisely identified the manually defined CA2 area without RGS14 immunofluorescence (Figures 3, 4), possibly by capturing some characteristics of cytoarchitecture in the CA2 area. In this sense, CAseg may be comparable to skilled experimenters in terms of segmentation of the CA2 area.

RGS14 has recently been used as a marker protein of the CA2 pyramidal cells of mice (Lee et al., 2010; Noguchi et al., 2017; Alexander et al., 2019). However, it remained to be investigated whether prairie vole hippocampal neurons expressed RGS14; note that *Rgs14* is expressed in the medial preoptic area of the hypothalamus of adult male prairie voles (Seelke et al., 2018). Using immunohistochemical techniques, we found RGS14 expression in the vole hippocampus and surmised that the RGS14-positive area represented the CA2 subfield because the hippocampal pyramidal cell layer was explicitly segmented (Figure 5A). We then demonstrated that CAseg could be extended to prairie voles based on the fact that the performance metric for predicting the RGS14-positive area was not significantly different between prairie voles and mice (Figure 5B). Although we did not physiologically demonstrate that the RGS14-positive area in the vole hippocampus signified the CA2 area, performance of CAseg suggests that RGS14 can also be used as a marker protein for the vole CA2 area. Since the CA2 area has also been investigated in other rodent species such as rats (Benoy et al., 2021), guinea pigs (Bartesaghi and Severi, 2004), and gerbils (Hisamatsu et al., 2016), a modified form of CAseg would precisely identify the rodent CA2 area in Nissl-stained brain sections. While the neurons of primates such as monkeys and humans also densely express RGS14 in a certain subarea of the hippocampus, which might represent the CA2 area (Squires et al., 2018), extending capabilities of CAseg for such phylogenetically distant species is the future challenge. However, our automated segmentation algorithm, CAseg, opens a new door for neuroscientists to systematically isolate the rodent CA2 area and will help them examine its structural and functional specificity in the rodent hippocampus.

## Data availability statement

The original contributions presented in this study are included in the article/Supplementary material, further inquiries can be directed to the corresponding author.

## Ethics statement

The animal study was reviewed and approved by the animal experiment ethics committee at the University of Tokyo (approval numbers: P29–15 and P3-1).

## Author contributions

NM and YI conceived the research. YT, AN, and JL performed the experiments. YT, KY, and NM analyzed the data. SM provided the resources. All authors discussed the project and approved the final version of the manuscript.
