# Supplementary material for: Machine learning-based segmentation of the rodent hippocampal CA2 area from Nissl-stained sections
Source: Front Neuroanat. 2023 Jun 28;17:1172512. doi: 10.3389/fnana.2023.1172512 (PMC10336234; doi:10.3389/fnana.2023.1172512)
Supplement: Supplementary file 1 [file Data_Sheet_1.PDF]

Takeuchi *et al.*, Supplementary Materials

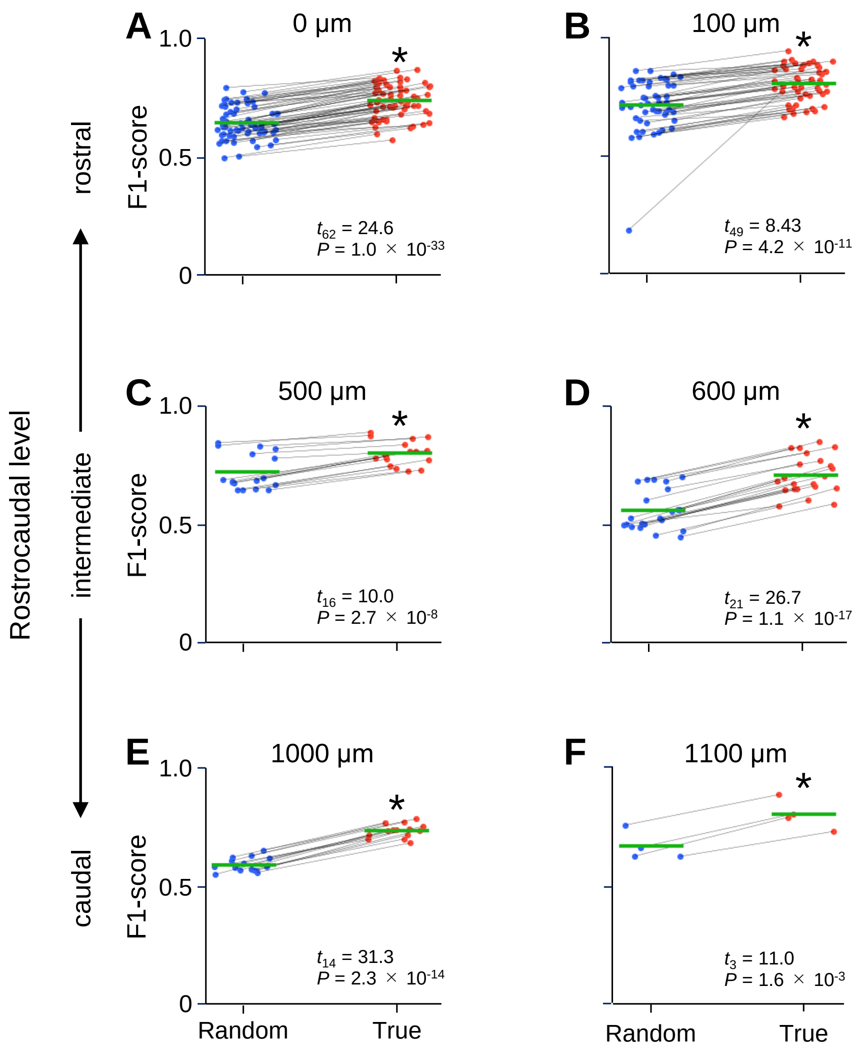

Supplementary Figure 1 (related to Figure 4) |
Performance metrics of CAseg for sections at different rostrocaudal levels.
The sections were grouped into the rostral (0 µm, 100 µm), intermediate (500 µm, 600 µm), and caudal (1000 µm, 1100 µm) levels. The CAseg performance on the three levels was then analyzed for random or true labels; note that ‘0 µm’ indicates the coronal sections (*i.e.*, test images) where the CA2 area was first found when the brain was cut from the anterior side. ***A***, F1-score of CAseg with a 30% threshold and one iteration. The test images (*i.e.*, coronal sections at 0 µm) were predicted by CAseg trained on random labels (*blue*) and true labels (*red*). Each pair of points connected by a line (*black*) signifies the same test image. The average F1-score calculated from the test data is shown in *green*. ***B-F***, The same as *A*, but for coronal sections at 100, 500, 600, 1000, 1100 µm, respectively. Statistics are compiled by paired *t*-tests.

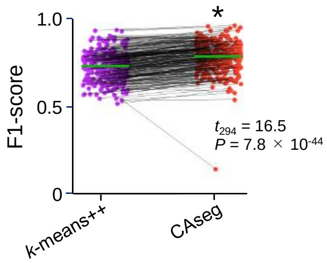

Supplementary Figure 2 (related to Figure 4) |
Segmentation performance metrics of *k*-means++ alone and CAseg.
The segmentation of the CA2 area was performed using *k*-means++ alone (*purple*) and CAseg (*red*). The F1-score was calculated in both cases. Statistics are compiled by a paired *t*-test.

Figure 4				Figure 5			
# of images	Side	Level (μm)	Mouse ID	# of images	Side	Level (μm)	Mouse ID
17	R	1200	1	1	R	1300	4
34	L	1200	1	1	R	1100	5
21	R	1300	1	1	R	600	6
20	L	1300	1	1	R	1200	17
15	L	1400	1	1	R	1400	17
28	R	400	2	1	L	700	21
17	R	1000	2	1	R	900	21
19	R	1100	7	1	L	1100	21
6	L	900	8	1	R	1200	21
13	R	800	14				
11	R	900	14				
19	R	400	15				
18	R	500	17				
16	L	400	19				
12	R	500	19				
5	R	1000	19				
4	L	1500	19				
8	R	500	21				
12	L	500	21				

‘Level (μm)’ indicates the rostrocaudal level.
Abbreviations: R, right; L, left.

Supplementary Table 1 (related to Figure 4 and 5) |
Information of the images of the mouse hippocampus used for CAseg.

Takeuchi *et al.*, Supplementary Materials

Parameter		Precision			
Threshold	Iteration(s)	Random label	True label	P	t_{294}
20%	0	0.51 ± 0.20	0.78 ± 0.12	3.7×10^{-74}	24.8
20%	1	0.51 ± 0.20	0.77 ± 0.12	3.4×10^{-73}	24.6
20%	2	0.50 ± 0.19	0.76 ± 0.12	6.6×10^{-72}	24.2
20%	3	0.50 ± 0.19	0.75 ± 0.12	1.9×10^{-70}	23.8
30%	0	0.58 ± 0.11	0.80 ± 0.12	2.4×10^{-172}	62.8
30%	1	0.57 ± 0.11	0.79 ± 0.12	3.9×10^{-171}	62.1
30%	2	0.57 ± 0.10	0.79 ± 0.12	1.6×10^{-169}	61.3
30%	3	0.56 ± 0.10	0.78 ± 0.12	5.8×10^{-168}	60.5
40%	0	0.60 ± 0.11	0.82 ± 0.11	1.5×10^{-172}	62.9
40%	1	0.59 ± 0.10	0.82 ± 0.11	6.5×10^{-174}	63.6
40%	2	0.59 ± 0.10	0.81 ± 0.11	3.4×10^{-174}	63.8
40%	3	0.58 ± 0.10	0.80 ± 0.11	8.4×10^{-174}	63.6
50%	0	0.63 ± 0.12	0.83 ± 0.11	1.8×10^{-146}	50.3
50%	1	0.62 ± 0.12	0.83 ± 0.11	8.6×10^{-150}	51.8
50%	2	0.61 ± 0.12	0.82 ± 0.11	4.2×10^{-152}	52.8
50%	3	0.61 ± 0.11	0.82 ± 0.11	4.8×10^{-153}	53.3

Statistics are compiled by paired *t*-tests.

Supplementary Table 2 (related to Figure 4) |
Precision for CAseg trained on random and true labels.

Parameter		Recall			
Threshold	Iteration(s)	Random label	True label	*P*	t_{294}
20%	0	0.75 ± 0.22	0.79 ± 0.13	5.0×10^{-4}	3.5
20%	1	0.75 ± 0.22	0.80 ± 0.13	1.3×10^{-4}	3.9
20%	2	0.75 ± 0.22	0.80 ± 0.13	5.4×10^{-5}	4.1
20%	3	0.75 ± 0.22	0.81 ± 0.13	3.0×10^{-5}	4.2
30%	0	0.81 ± 0.13	0.77 ± 0.12	1.5×10^{-25}	-11.5
30%	1	0.81 ± 0.13	0.78 ± 0.13	9.3×10^{-19}	-9.5
30%	2	0.82 ± 0.13	0.79 ± 0.13	4.2×10^{-14}	-7.9
30%	3	0.82 ± 0.13	0.80 ± 0.13	3.9×10^{-11}	-6.9
40%	0	0.81 ± 0.12	0.74 ± 0.12	1.2×10^{-63}	-21.8
40%	1	0.82 ± 0.12	0.76 ± 0.12	8.7×10^{-56}	-19.7
40%	2	0.82 ± 0.12	0.77 ± 0.12	1.9×10^{-49}	-18.0
40%	3	0.82 ± 0.12	0.77 ± 0.12	9.9×10^{-45}	-16.8
50%	0	0.81 ± 0.13	0.70 ± 0.12	1.4×10^{-55}	-19.7
50%	1	0.81 ± 0.13	0.72 ± 0.12	1.0×10^{-49}	-18.1
50%	2	0.81 ± 0.13	0.73 ± 0.12	2.9×10^{-44}	-16.6
50%	3	0.82 ± 0.13	0.74 ± 0.12	5.6×10^{-40}	-15.5

Statistics are compiled by paired *t*-tests.

Supplementary Table 3 (related to Figure 4) |
Recall for CAseg trained on random and true labels.

Parameter		F1-score			
Threshold	Iteration(s)	Random label	True label	P	t_{294}
20%	0	0.59 ± 0.21	0.77 ± 0.09	2.9×10^{-39}	15.3
20%	1	0.59 ± 0.21	0.77 ± 0.09	1.3×10^{-39}	15.4
20%	2	0.59 ± 0.21	0.77 ± 0.09	1.7×10^{-39}	15.4
20%	3	0.59 ± 0.20	0.77 ± 0.09	3.8×10^{-39}	15.3
30%	0	0.67 ± 0.09	0.78 ± 0.09	4.6×10^{-105}	34.4
30%	1	0.66 ± 0.09	0.78 ± 0.09	5.4×10^{-109}	35.7
30%	2	0.66 ± 0.09	0.78 ± 0.09	4.0×10^{-111}	36.5
30%	3	0.66 ± 0.09	0.78 ± 0.09	7.5×10^{-112}	36.7
40%	0	0.68 ± 0.08	0.77 ± 0.08	1.2×10^{-97}	32.0
40%	1	0.68 ± 0.08	0.78 ± 0.08	1.5×10^{-106}	34.9
40%	2	0.67 ± 0.08	0.78 ± 0.08	1.0×10^{-113}	37.4
40%	3	0.67 ± 0.08	0.78 ± 0.08	6.3×10^{-119}	39.3
50%	0	0.70 ± 0.09	0.75 ± 0.08	1.5×10^{-34}	14.0
50%	1	0.69 ± 0.09	0.76 ± 0.08	1.5×10^{-45}	17.0
50%	2	0.69 ± 0.09	0.76 ± 0.08	7.3×10^{-56}	19.8
50%	3	0.69 ± 0.09	0.77 ± 0.08	9.7×10^{-65}	22.2

Statistics are compiled by paired *t*-tests.

Supplementary Table 4 (related to Figure 4) |
F1-score for CAseg trained on random and true labels.

Takeuchi *et al.*, Supplementary Materials

**Parameter**		**IoU**			
Threshold	Iteration(s)	Random label	True label	*P*	t_{294}
20%	0	0.45 ± 0.18	0.64 ± 0.12	1.5×10^{-54}	19.4
20%	1	0.44 ± 0.17	0.64 ± 0.12	7.4×10^{-55}	19.5
20%	2	0.44 ± 0.17	0.64 ± 0.12	1.4×10^{-54}	19.4
20%	3	0.44 ± 0.17	0.63 ± 0.11	5.8×10^{-54}	19.2
30%	0	0.51 ± 0.10	0.64 ± 0.11	8.1×10^{-112}	36.7
30%	1	0.50 ± 0.10	0.65 ± 0.12	3.0×10^{-115}	38.0
30%	2	0.50 ± 0.10	0.65 ± 0.12	6.5×10^{-117}	38.5
30%	3	0.50 ± 0.10	0.64 ± 0.12	1.6×10^{-117}	38.7
40%	0	0.52 ± 0.10	0.63 ± 0.11	8.1×10^{-97}	31.7
40%	1	0.52 ± 0.10	0.64 ± 0.11	5.7×10^{-105}	34.4
40%	2	0.51 ± 0.10	0.64 ± 0.11	2.2×10^{-111}	36.6
40%	3	0.51 ± 0.09	0.64 ± 0.11	7.9×10^{-116}	38.1
50%	0	0.54 ± 0.11	0.61 ± 0.11	1.2×10^{-37}	14.9
50%	1	0.54 ± 0.11	0.62 ± 0.11	1.9×10^{-49}	18.0
50%	2	0.54 ± 0.11	0.63 ± 0.11	2.2×10^{-60}	21.0
50%	3	0.53 ± 0.11	0.63 ± 0.11	1.2×10^{-69}	23.5

Statistics are compiled by paired *t*-tests.

Supplementary Table 5 (related to Figure 4) |
IoU (intersection over union) for CAseg trained on random and true labels.
